# STAT-Mediated Mitochondrial Regulation in Cardiovascular Diseases: Mechanistic Insights and STAT3-Focused Therapeutic Strategies

**DOI:** 10.3390/biom16020286

**Published:** 2026-02-11

**Authors:** Bing Guo, Yan Fu, Min Wang, Lemei Zhu, Xuan He

**Affiliations:** 1 Graduate School, Hunan University of Chinese Medicine, Changsha 410208, China; 20203219@stu.hnucm.edu.cn (B.G.); 20232019@stu.hnucm.edu.cn (M.W.); 2 Medical College, Hunan University of Chinese Medicine, Changsha 410208, China; 202208010312@stu.hnucm.edu.cn; 3 School of Public Health, Changsha Medical University, Changsha 410219, China; 4 Hunan Key Laboratory of the Research and Development of Novel Pharmaceutical Preparations, Changsha Medical University, Changsha 410219, China; 5 College of Traditional Chinese Medicine, Changsha Medical University, Changsha 410219, China

**Keywords:** signal transducer and activator of transcription (STAT), mitochondrial function, cardiovascular disease, pharmacological treatment

## Abstract

Mitochondria, the cell’s powerhouses, generate ATP to sustain essential biological functions. Dysfunctional mitochondria can lead to cell death and subsequent tissue damage. Mitochondrial impairment is a key driver of cellular dysfunction in cardiomyocytes, endothelial cells, and macrophages, contributing to cardiovascular diseases such as atherosclerosis, myocardial ischemia–reperfusion injury, and cardiac hypertrophy. The signal transducer and activator of transcription (STAT) family regulates immune responses, apoptosis, and cell proliferation. Despite evidence suggesting that STATs influence mitochondrial pathways in various cardiovascular conditions, their roles are often contradictory and context-dependent. This review examines the structural and functional dynamics of STATs, their upstream and downstream signaling networks, and therapeutic strategies targeting STAT3 (the most extensively studied isoform), with a particular focus on natural compounds and pharmacological inhibitors. By synthesizing current findings, this review offers valuable insights into STATs as potential therapeutic targets for mitochondrial dysfunction in cardiovascular diseases, while also highlighting directions for future research.

## 1. Introduction

Cardiovascular diseases (CVDs) remain the leading cause of death and morbidity worldwide. The global prevalence of CVDs nearly doubled, increasing from 271 million in 1990 to 523 million in 2019. In parallel, the number of deaths attributable to CVDs rose steadily, from 12.1 million in 1990 to 18.6 million in 2019 [1]. This significant burden presents substantial socioeconomic challenges, making the exploration of underlying mechanisms and potential treatments for CVDs a research priority.

CVDs encompass a wide range of conditions, including ischemia–reperfusion (I/R) injury, heart failure, cardiac hypertrophy, and atherosclerosis [2]. Key mechanisms driving these diseases involve inflammation [3], oxidative stress [4], and mitochondrial dysfunction [5]. Mitochondria are particularly critical due to their role in ATP production, which fuels cardiomyocytes [6]. They are also major sources of reactive oxygen species (ROS) under oxidative stress [7]. Impaired mitochondrial function can trigger cardiomyocyte death, ultimately compromising heart function [8].

The progression of CVDs is also associated with alterations in gene expression and signaling pathways. Recent evidence highlights the pivotal role of the signal transducer and activator of transcription (STAT) protein family in CVD pathogenesis. STAT proteins are expressed in various cardiac cells, including cardiomyocytes and fibroblasts, where they regulate vital processes such as cell death, angiogenesis, and inflammation [9]. While previous studies and reviews have highlighted the involvement of STAT signaling in CVDs at the level of global signaling pathways [9,10], their roles in mitochondrial regulation have received comparatively limited attention.

In contrast, this review focuses on mitochondrial dysfunction as a central pathological feature of CVDs and systematically summarizes the regulatory roles of STAT family members in mitochondrial function across different cardiovascular conditions. In addition, we emphasize recent advances in targeting the *STAT3*–mitochondrial signaling axis as a potential therapeutic strategy. Specifically, we discuss pharmacological approaches, including natural compounds, specific inhibitors, and conventional drugs, that modulate STAT signaling to improve mitochondrial function. Overall, this review aims to provide new mechanistic insights into STAT-mediated mitochondrial regulation and to highlight the therapeutic potential of targeting STAT proteins, particularly *STAT3*, for the prevention and treatment of CVDs.

## 2. Search Strategy

Relevant articles published within the past 20 years were retrieved from the PubMed and Web of Science databases using search terms related to STATs, mitochondria, and cardiovascular diseases. Only full-text articles published in English were considered. The search results were further screened based on titles and abstracts, and only studies focusing on the mechanisms by which STATs mediate mitochondrial regulation in cardiovascular diseases were included. The search terms were as follows: “cardiovascular diseases”, “atherosclerosis”, “septic cardiomyopathy”, “myocardial infarction”, “heart failure”, “mitochondrial dynamics”, “mPTP”, “mitochondrial apoptosis”, “myocardial ischemia–reperfusion injury”, “mitochondria”, “doxorubicin-induced cardiomyopathy”, “STAT”, “heart disease”, “therapy”, “natural compounds” and “clinical drugs”.

## 3. The STATs Family

### 3.1. Structure and Function of STATs and Mitochondria

**Figure 1:**
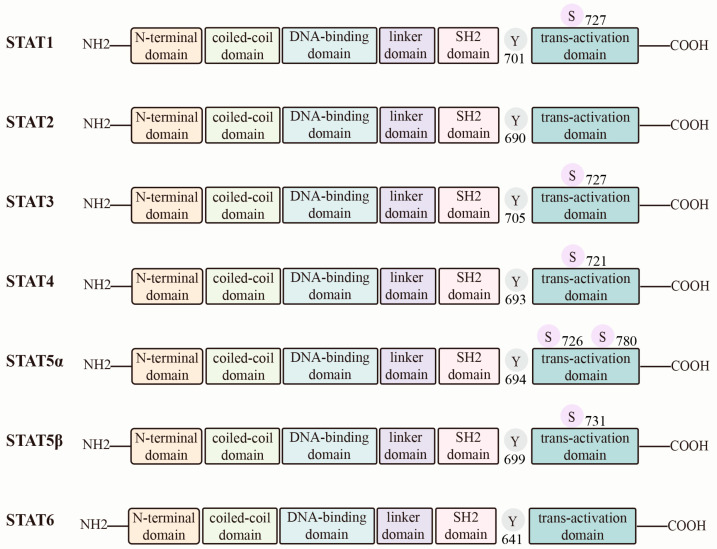
The structure of STAT family protein. STAT, signal transducer and activator of transcription. This figure was created by the authors using Figdraw (www.figdraw.com).

The STAT (signal transducer and activator of transcription) family comprises seven members: *STAT1*, *STAT2*, *STAT3*, *STAT4*, *STAT5A*, *STAT5B*, and *STAT6* [11]. These proteins are central mediators of cellular signal transduction. They regulate gene expression and thereby influence fundamental processes such as cell proliferation, differentiation, and apoptosis. All STAT proteins share conserved domains, including the N-terminal domain (NTD), coiled-coil domain (CCD), DNA-binding domain (DBD), linker domain (LD), Src homology 2 (SH2) domain, and transactivation domain (TAD) [12]. Notably, the SH2 domain binds to Janus kinase (JAK)-phosphorylated sites. This interaction promotes STAT dimerization, which is a prerequisite for canonical STAT signaling [13] (Figure 1).

Mitochondria exhibit a highly organized architecture that is maintained by the coordinated function of the outer mitochondrial membrane (OMM) and inner mitochondrial membrane (IMM) [14]. The OMM contains porous channels that permit the passage of small molecules and ions [15]. This permeability facilitates metabolite transport and supports substrate exchange required for ATP production and other metabolic pathways [16]. In contrast, the IMM is extensively folded into cristae. These structures provide the platform for oxidative phosphorylation (OXPHOS) [17] and enable the proton gradient essential for ATP synthesis [18]. The mitochondrial electron transport chain is the core of eukaryotic energy metabolism. It consists of four respiratory complexes. Together, these complexes generate an electrochemical gradient that drives ATP synthase activity [19]. Beyond structural organization, mitochondrial function is also shaped by dynamic remodeling. Fusion and fission determine mitochondrial morphology and metabolic adaptability [20]. Therefore, mitochondrial homeostasis relies on both membrane architecture and dynamic balance.

Recent studies indicate that STAT proteins function beyond their nuclear role as transcription factors. Several STATs also exert regulatory activities within mitochondria, with STAT3 being a prominent example. Phosphorylation of *STAT3* at serine 727 enhances its mitochondrial actions and does not require nuclear localization [21]. Mitochondrial *STAT3* (mtSTAT3) promotes ATP production and reduces reactive oxygen species (ROS) generation [22]. However, the underlying mechanisms remain incompletely defined. Accordingly, the following sections will summarize how STATs modulate mitochondrial function in cardiovascular diseases, with a particular focus on STAT3-dependent mitochondrial regulation and emerging therapeutic strategies targeting the *STAT3*–mitochondria axis.

### 3.2. STATs Upstream Activation Mechanism

**Figure 2:**
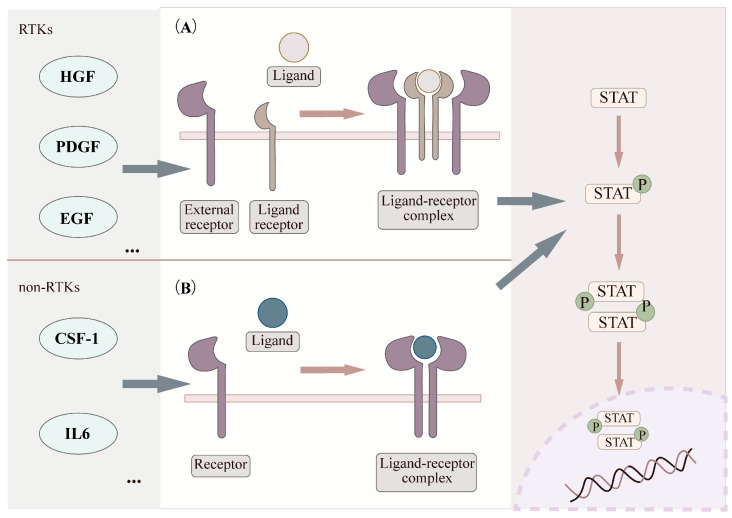
The activation mechanism of STAT proteins. (**A**) Activation of the STAT pathway via RTKs. Ligand binding to RTKs induces the formation of a ligand-receptor complex, which directly phosphorylates STAT proteins. (**B**) STAT activation through non-RTKs. Ligand binding to non-RTKs, such as Src or bcr-abl, indirectly activates STAT proteins by phosphorylation. STAT, signal transducer and activator of transcription; RTKs, receptor tyrosine kinases. This figure was created by the authors using Figdraw (www.figdraw.com).

The STAT signaling pathway is activated by various extracellular signals, including growth factors such as hepatocyte growth factor (*HGF*) [23], platelet-derived growth factor (*PDGF*) [24], epidermal growth factor (*EGF*) [25], and colony-stimulating factor 1 (*CSF-1*) [26] as well as cytokines like interleukin-6 (*IL-6*) [27]. STAT activation primarily occurs through two mechanisms. The first mechanism involves receptor tyrosine kinases (RTKs). Upon ligand binding, RTKs initiate signaling and can directly phosphorylate STAT proteins due to their intrinsic tyrosine kinase activity. Alternatively, RTKs can activate non-RTKs, such as JAKs, which subsequently phosphorylate STATs [28]. The second mechanism involves direct activation by non-RTKs, including kinases like Src and bcr-abl, which interact with and directly phosphorylate STAT proteins [29,30]. Furthermore, evidence suggests that G protein-coupled receptors (GPCRs) can also contribute to STAT activation. Notable examples include receptors for angiotensin II human (*Ang II*) and 5-hydroxytryptamine receptor 2A (*5-HT2A*) [31,32]. These upstream events lead to the phosphorylation of specific tyrosine residues on STATs. Phosphorylation triggers STAT dimerization. The dimerized STATs then translocate into the nucleus, where they regulate the transcription of target genes [33] (Figure 2).

However, current studies largely focus on the classical pathway, where STATs translocate to the nucleus to regulate gene expression, while the potential roles of STATs in the cytoplasm or mitochondria remain largely overlooked. As the role of molecules such as *STAT3* in mitochondrial function becomes increasingly recognized, it is crucial to reassess whether this signaling pathway also regulates mitochondrial function, morphology, and metabolic activities to influence cellular energy production and redox status. Given the importance of mitochondria in both physiological and pathological contexts, it is essential to expand research beyond the nucleus and focus more on how STATs modulate mitochondrial function and their potential impact on cardiovascular diseases.

## 4. The STAT Family and Mitochondria in Cardiovascular Diseases

### 4.1. STATs Protein Localize to the Mitochondria

Early research primarily characterized STAT proteins as mediators of cytoplasm-to-nucleus signaling [34]. However, recent evidence reveals their presence in other subcellular compartments, particularly mitochondria [35]. Within mitochondria, STATs influence mitochondrial protein activity and cellular metabolism by modulating the mitochondrial electron transport chain (ETC) and the mitochondrial permeability transition pore (mPTP), thus contributing to the maintenance of cellular energy balance [36,37,38]. Multiple studies have confirmed that STAT proteins localize to mitochondria and perform distinct functions. For example, STAT1 is detected in the mitochondria of cardiomyocytes. In cardiomyocytes, mitochondrial *STAT1* lowers the mitochondrial membrane potential (ΔΨm) and reduces mitochondrial density [39]. For *STAT2*, research by Goswami et al. suggests that viral infection can trigger its mitochondrial localization, potentially as a mechanism to suppress the antiviral response [40]. However, whether it regulates mitochondrial pathways in viral myocarditis has not yet been investigated. Another study demonstrated that *STAT2* promotes macrophage differentiation by enhancing mitochondrial quality through a dynamin-related protein 1 (*Drp1*)-dependent pathway [41]. However, the general applicability of this mechanism across cardiovascular diseases, as well as its potential crosstalk with other signaling pathways, has not yet been sufficiently validated. Therefore, future studies should further investigate the regulatory mechanisms of this pathway in diverse cardiovascular disease models, such as atherosclerosis, to establish its reliability and therapeutic relevance in clinical settings.

Among the most extensively studied STAT family members, STAT3 translocates to mitochondria in various cell types, including macrophages [42] and cardiomyocytes [43]. In addition to regulating basic mitochondrial functions, *STAT3* participates in the regulation of multiple cell death pathways, such as autophagy [44], necroptosis [45] and apoptosis [46]. In contrast, no direct evidence yet places *STAT4* within mitochondria, highlighting a notable gap in current understanding. For *STAT5*, studies show that mitochondrial superoxide dependence enhances its chromatin accessibility, promoting the transformation of macrophages into foam cells and exacerbating atherosclerosis [47]. However, these findings primarily reflect indirect mitochondrial influences on *STAT5* activity rather than direct mitochondrial localization or mechanistic action. Similarly, under hypoxic conditions, OMM–associated *STAT6* inhibits mitochondrial fusion through disruption of MFN2 dimerization, suggesting that STAT6 serves as a regulator of mitochondrial processes and contributes to mitochondrial dynamic imbalance [48]. Nevertheless, whether this mechanism operates in a cell type or cardiovascular disease-specific manner remains to be determined, and the broader relevance of *STAT6*-mediated mitochondrial regulation under physiological and pathological conditions warrants further investigation.

In summary, various STAT family members localize to mitochondria, where they regulate mitochondrial function and cellular energy metabolism. Given this role, STAT proteins are poised to significantly influence the pathogenesis of CVDs. The following sections explore this regulatory interplay in more detail.

### 4.2. STAT1

*STAT1* plays a central role in inflammation and cellular stress responses. Its function is regulated by phosphorylation, upstream cytokines, and subcellular localization. In chronic Chagas cardiomyopathy, heart samples from patients and AC16 cardiomyocyte models show severe mitochondrial damage, including reduced membrane potential, loss of mtDNA, and bursts of ROS. The *IFN-γ*/*TNF-α*-activated *STAT1*/*NF-κB*/*NOS2* pathway is considered a key mechanism driving these defects [49]. However, the specific phosphorylation sites involved in *STAT1*’s action were unclear until further investigation. Z-DNA binding protein 1 (*ZBP1*) has been shown to recognize and stabilize the Z-form of mitochondrial DNA, stabilizing *STAT1* phosphorylation at S727. This drives the type I interferon response and exacerbates inflammatory injury in cardiomyocytes [50]. Moreover, highly spiraled mtDNA enhances *STAT1* S727 phosphorylation [51], suggesting that the mitochondrial genome is not just a passive target but also an upstream regulator of STAT1 signaling. Thus, the role of *STAT1* extends beyond that of a nuclear transcription factor to include functioning as a sensor of mitochondrial stress.

In pulmonary hypertension, *STAT1* regulates mitochondrial dynamics by modulating MMP8 and upregulating Drp1, promoting excessive mitochondrial fission in pulmonary arterial endothelial cells. This, in turn, drives endothelial proliferation and vascular remodeling [52]. In contrast, *STAT1* exhibits a different effect in heart hypertrophy. *STAT1*-deficient mice show more pronounced hypertrophy and fibrosis. In cardiomyocytes, phosphorylated *STAT1* at Y701 promotes Drp1 expression and mitochondrial fission, increasing compensatory ATP production while downregulating hypertrophy-related genes such as atrial natriuretic peptide (*ANP*), b-type natriuretic peptide (*BNP*), and myosin heavy chain (*MHC)* [53]. These contrasting effects likely arise from cell type-specific functions of *STAT1*. However, its role in promoting mitochondrial fission remains consistent. Furthermore, in cardiomyocyte hypertrophy, STAT1 also induces mitochondrial dysfunction and cytochrome c release, leading to cardiomyocyte apoptosis [39]. The apparent contradiction in STAT1’s role in cardiac hypertrophy may be linked to its phosphorylation status, with different disease stages potentially influencing its function. Further studies are required to clarify these mechanisms.

*STAT1* exhibits a clear “double-edged sword” effect in myocardial I/R injury. During the late ischemic phase, nuclear phosphorylation of *STAT1* at Y701 upregulates the anti-apoptotic protein Mcl-1, offering protection to cardiomyocytes [54]. However, another study reveals that *STAT1* can also localize directly to mitochondria and translocate to cytoplasmic autophagosomes, exacerbating damage in primary cardiomyocytes and fibroblasts [55]. This discrepancy may be related to *STAT1*’s subcellular location and the disease state. Nuclear *STAT1* typically regulates anti-apoptotic genes, while mitochondrial *STAT1* directly influences mitochondrial phenotypes. This location-dependent effect helps explain the “spatiotemporal effect” of *STAT1*.

In immune cells, *STAT1* is also linked to mitochondria. For example, using mitochondrial ETC inhibitors in macrophages induces mitochondrial dysfunction, leading to interferon-induced *STAT1* phosphorylation at Y701. This activates Cxcl9/10 expression and promotes inflammation [56]. Thus, *STAT1* acts as a key mediator connecting inflammatory signals with mitochondrial pathology.

In summary, *STAT1* primarily functions in cardiomyocytes, pulmonary arterial endothelial cells, cardiac fibroblasts, and macrophages. Through phosphorylation at S727 and Y701, it interacts with mtDNA and regulates mitochondrial dynamics to maintain mitochondrial quality. This regulation is bidirectional and may depend on cell type, phosphorylation status, and disease stage. Further investigation is necessary to better understand these complex mechanisms.

### 4.3. STAT3

STAT3 is the most functionally complex member of the STAT family, with most research suggesting that it exerts protective effects through mitochondria. The mechanism typically involves *STAT3* translocating to cardiomyocyte mitochondria, where it is phosphorylated at both Y701 and S727. This process regulates the ETC, mitochondrial respiration, calcium homeostasis, and mPTP activity, thereby promoting cardiomyocyte survival [57,58,59,60,61]. However, *STAT3*’s regulation of mitochondria exhibits dual roles across different diseases.

In myocardial I/R injury, dilated cardiomyopathy (DCM), and peripartum cardiomyopathy, STAT3 plays a beneficial role. In myocardial I/R injury, *STAT3* is regulated by various signals and organelles. In endothelial cells, endoplasmic reticulum stress inhibits mitochondrial *STAT3* phosphorylation at S727, which promotes mitochondrial calcium overload, ROS production, and mPTP formation [62]. Several protective molecules, such as *IL-35*, *HDL*, *IL-11*, and *CXCR4*, activate *STAT3* to enhance mitochondrial function, thus mitigating I/R injury [63,64,65,66]. However, while these findings suggest promising therapeutic avenues, the question remains whether these protective effects can be reliably replicated across different disease models and cell types. Furthermore, the temporal dynamics of *STAT3* activation during I/R injury are largely unexplored, and how early vs. late activation of *STAT3* influences the outcome is still unclear. Notably, during early reperfusion in rats, ROS can be protective by activating the *JAK2/STAT3* pathway to upregulate *Bcl2*, maintaining calcium homeostasis and improving mitochondrial function [66]. However, this protective role of ROS in early reperfusion must be interpreted with caution, as excessive ROS generation later in reperfusion is often associated with detrimental effects, such as oxidative damage and cellular apoptosis. Therefore, while ROS and STAT3 activation may offer protective benefits in specific windows of time.

In DCM, reduced levels of *MFF* and *STAT3* are observed in cardiac endothelial cells in rabbit plasma, which coincides with impaired endothelial barrier function [67]. Further mechanistic studies show that glycoprotein 130 (*Gp130*) promotes *STAT3* phosphorylation at Y705, counteracting mitochondrial dysfunction and intracellular Ca^2+^ overload, thereby reducing doxorubicin-induced cardiomyocyte apoptosis and oxidative stress [68]. Calreticulin (CRT), an endoplasmic reticulum chaperone that binds calcium and localizes to mitochondria, impairs cytochrome c levels and reduces *STAT3* phosphorylation at Y705 in mitochondrial fractions. This exacerbates cardiomyocyte apoptosis [69,70]. Conversely, heat shock protein 22 (*HSP22*) promotes *STAT3* translocation from the nucleus to mitochondria, increasing its phosphorylation at Y705. This enhances mitochondrial respiration and inhibits oxidative stress [71,72]. These findings demonstrate that *STAT3* improves DCM symptoms through mitochondrial phosphorylation.

In peripartum cardiomyopathy, *STAT3* deficiency leads to excessive mitochondrial oxidative stress and dysfunction [73]. A similar phenomenon is observed in diabetic cardiomyopathy, where overactivation of Forkhead box O1 (*FOXO1*) inhibits *STAT3* phosphorylation. This promotes *Drp1* expression and suppresses MFN protein levels, disrupting mitochondrial dynamics, increasing ROS production, reducing ATP, and decreasing cardiac ejection fraction in mice [74].

However, in cardiac hypertrophy, *STAT3*’s regulation of mitochondria is detrimental. Dual-specificity tyrosine phosphorylation-regulated kinase 1B (*DYRK1B*) directly binds *STAT3*, promoting its phosphorylation at Y705 and nuclear accumulation. This impairs mitochondrial energy metabolism, inhibits peroxisome proliferator-activated receptor γ coactivator-1α (*PGC-1α*) expression, and disrupts mitochondrial biogenesis, contributing to cardiac hypertrophy [43]. Increased levels of carnitine palmitoyltransferase 1 (*CPT1*) and fatty acid oxidation (*FAO*) promote *STAT3* phosphorylation at Y705 and nuclear translocation, inducing lipid overload-associated mitochondrial dysfunction and cardiac hypertrophy [75]. In human fibroblasts, the nuclear receptor retinoic acid receptor (RAR)-related orphan receptor α (*RORα*) inhibits the *IL-6/STAT3* pathway, reducing Y705 phosphorylation and nuclear accumulation. This enhances mitochondrial number and function, alleviating AngII-induced pathological hypertrophy [76]. These studies suggest that *STAT3* accumulation in the nucleus may mediate its adverse effects in hypertrophy. However, the specific mechanism of *STAT3* movement from the nucleus to mitochondria remains unclear. Additionally, the activated phosphorylation site (Y705) in hypertrophy differs from those in I/R injury (Y701/S727), which may also explain the differing effects. Future research should focus on the “nucleus-mitochondria” crosstalk of *STAT3* in hypertrophy.

In septic cardiomyopathy, *STAT3* promotes inflammation and mitochondrial injury. *PPARα* deletion activates the *IL-6/STAT3/NF-κB* axis, exacerbating ROS production and mitochondrial dysfunction [77,78].

In summary, different disease states create distinct cellular environments that affect STAT3 phosphorylation and its distribution between the nucleus and mitochondria. For instance, in septic cardiomyopathy, robust inflammation may favor nuclear *STAT3*, promoting oxidative stress, while in DCM, the focus is on mitochondrial STAT3 phosphorylation. Future research should systematically examine how *STAT3*’s nuclear/mitochondrial distribution changes, particularly during the early and late stages of disease.

### 4.4. Other STATs

Other STAT members primarily respond to immune cells and signals, influencing CVDs by regulating mitochondrial dynamics and quality control.

*STAT6* exhibits dual roles in CVDs. In atherosclerosis, *STAT6* expression can mitigate the disease, potentially by promoting macrophage polarization to the M2 anti-inflammatory phenotype, which reduces lipid accumulation [79,80]. Blocking ROS with mitoTEMPO inhibits *STAT6* activation and reduces M2 polarization [81]. Additionally, interleukin-4 (*IL-4*) promotes *STAT6* phosphorylation, enhancing *PGC-1β* expression and inducing mitochondrial biogenesis in macrophages, thereby fostering anti-inflammatory macrophage maturation [82].

However, in sepsis and LPS-induced models, the *JAK2/STAT6* pathway promotes oxidative stress, increasing ROS levels, which leads to cardiomyocyte apoptosis and cardiac dysfunction [83]. This study did not clarify whether *STAT6* affects ROS generation by localizing to mitochondria, necessitating further investigation.

Upon LPS stimulation, *STAT2* expression is significantly upregulated in macrophages, promoting mitochondrial fission and inducing macrophage differentiation into a pro-inflammatory phenotype, which increases ROS production [41]. In DCM, *STAT2* works with *STAT1* in the mtDNA damage-triggered type I interferon response [51], suggesting that *STAT2* plays a role in immune regulation, potentially contributing to cardiovascular pathology.

*STAT4* may promote vascular smooth muscle cell (VSMC) proliferation. Phosphorylation at S693 inhibits cytochrome c release and *Caspase-3* activation, suppressing VSMC apoptosis via the mitochondrial apoptosis pathway. This action promotes neointimal regeneration, leading to restenosis after arterial intervention [84].

Research on *STAT5* remains limited, but one study indicates that in a myocardial ischemia model, *STAT5* phosphorylation at Y694 inhibits ischemia-induced mitochondrial ROS production, boosts antioxidant enzyme expression, and inhibits fibroblast apoptosis, suggesting therapeutic potential [85].

**Figure 3:**
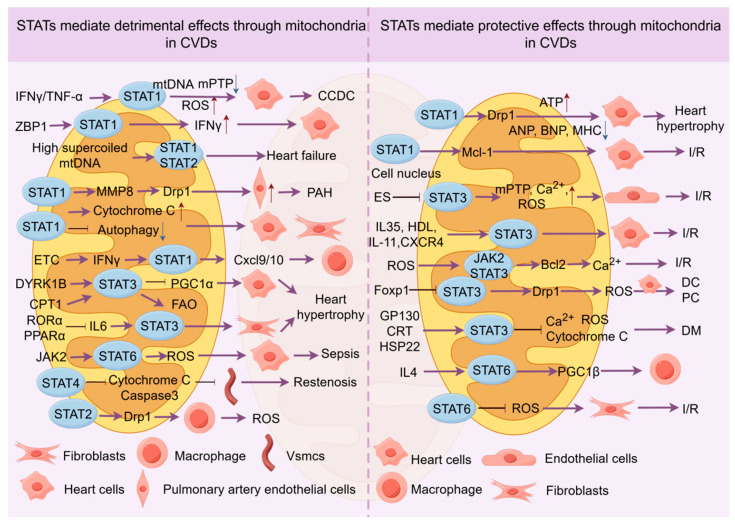
STAT proteins mediate mitochondrial mechanisms in cardiovascular disease. This image introduces how STAT proteins primarily mediate mitochondrial ROS, Ca^2+^, mPTP, autophagy, MMP, mtDNA, etc., in various cardiovascular diseases, thereby affecting mitochondrial function. In addition, STAT proteins also form a complex network with other molecules to jointly regulate mitochondrial function. STAT, signal transducer and activator of transcription; ROS, reactive oxygen species; mPTP, mitochondrial permeability transition pore; MMP, mitochondrial membrane potential; The upward arrow (↑) indicates an increase, whereas the downward arrow (↓) indicates a decrease. This figure was created by the authors using Figdraw (www.figdraw.com).

In conclusion, research on STATs other than *STAT1* and *STAT3* is still in its early stages. Most studies suggest these proteins may regulate mitochondrial function, but their potential dual roles in different cell types and their upstream mechanisms remain unclear. Further investigation is necessary to better understand these proteins’ roles in CVDs (Figure 3).

## 5. Therapeutic Approaches

There is growing interest in targeting *STAT3* to regulate mitochondrial function as a therapeutic strategy for treating CVDs. Current approaches primarily involve natural compounds and other pharmacological agents that modulate *STAT3* activity, influencing key mitochondrial parameters such as ROS production, malondialdehyde (MDA) levels, MMP, and mitochondrial dynamics. These treatments also impact the balance between fusion and fission, as well as apoptosis and respiratory function. Through these actions, the interventions aim to enhance cardiomyocyte function and improve cardiac outcomes (Table 1).

### 5.1. Natural Compounds

Recent studies have highlighted the potential of natural compounds that target the *STAT3*-mitochondria axis in CVDs. In atherosclerosis and acute myocardial infarction, esculetin, a natural derivative from Sophora japonica, reduces oxidative stress, inflammation, and apoptosis [86]. It acts on mitochondria, inhibits *STAT3* phosphorylation at Tyr705, lowers plasminogen activator inhibitor-1 activity, and attenuates inflammation [87]. Quercetin, a widely available flavonoid, possesses antioxidant, anti-platelet, and endothelial protective properties [88]. It improves outcomes in acute myocardial infarction by elevating p-STAT3 (Ser727) levels, suppressing ROS and MDA generation, limiting mPTP opening, and downregulating apoptosis-related proteins [89].

In doxorubicin-induced cardiomyopathy, flavonoids derived from fruits, vegetables, and medicinal plants exhibit strong cardioprotective effects in animal models, including reducing myocardial I/R injury [90]. Among them, the synthetic flavagline-inspired synthetic flavonoid (FL3) alleviates doxorubicin-induced cardiotoxicity by activating mitochondrial *STAT3* and reducing cardiomyocyte apoptosis [91]. 7,8-Dihydroxyflavone (7,8-DHF), found in plants such as *Godmania aesculifolia* and *Primula helleri* [92], helps restore MMP in injured cardiomyocytes. This compound promotes *STAT3* transcription, decreases ROS production, improves mitochondrial protein expression, and enhances ATP synthesis [93]. Animal studies confirm its protective role against doxorubicin-induced heart injury [94]. Paeoniflorin (PAE), a major bioactive compound from *Paeonia lactiflora*, exerts anti-inflammatory and antioxidant effects [95]. It activates *STAT3* and enhances mitochondrial fusion via mitofusin 2 (*Mfn2*), thereby counteracting doxorubicin-induced mitochondrial dysfunction and cardiac injury [96].

In diabetic cardiomyopathy, rosmarinic acid (RA), a polyphenol found in *Lamiaceae* plants, improves myocardial hypertrophy and increases ejection fraction [97]. RA restores mitochondrial function and reduces cardiomyocyte apoptosis by decreasing ROS generation, inhibiting mPTP activation, and preventing cytochrome c release and caspase-3 activation [98]. Taxifolin (TAX), a flavonoid isolated from Taxus species, also exhibits heart-protective effects. It improves mitochondrial function, reduces ROS, and alleviates diabetic cardiomyopathy by activating the JAK/STAT3 pathway [99]. TAX also enhances mitochondrial ATP production by inhibiting the *IL-6/JAK2/STAT3* pathway and reduces markers of myocardial hypertrophy, such as *BNP*, *ANP*, and *β-MHC* [100]. Furthermore, punicalagin, an ellagic acid component from pomegranate, promotes mitochondrial fusion by increasing *STAT3*/optic atrophy 1 (*Opa1*) expression. It reduces mitochondrial oxidative stress and cardiomyocyte apoptosis, thereby improving diabetic cardiomyopathy [101].

### 5.2. Clinical Drugs and Inhibitors

In cardiac hypertrophy, the lipid-lowering drug atorvastatin offers several cardioprotective benefits [102,103]. It suppresses *STAT3* phosphorylation, thereby reducing myocardial FAO. This mechanism improves mitochondrial function and mitigates lipid overload-induced cardiac hypertrophy [75]. Mild mitochondrial uncouplers also upregulate myocardial JAK/STAT3 expression, stimulate ATP production, and protect cardiomyocytes [104].

In I/R injury, zinc demonstrates therapeutic value for CVDs. Patients with heart failure frequently exhibit lower zinc levels [105]. Zinc supplementation enhances mitochondrial *STAT3* phosphorylation at Ser727 during reperfusion, improving oxidative phosphorylation and attenuating mitochondrial ROS generation [106]. Morphine alleviates myocardial I/R injury by promoting *STAT3* phosphorylation. It prevents MMP loss and ROS formation, preserves mitochondrial integrity, and suppresses cardiomyocyte autophagy [107]. Melatonin, with its pleiotropic effects, shows promise in I/R injury [108]. It activates *STAT3* phosphorylation, which increases mitochondrial superoxide dismutase (SOD) activity, reduces MDA and H_2_O_2_ production, and helps maintain redox balance, thereby protecting cardiomyocytes [109]. Intralipid, a parenteral nutrition source containing γ-linolenic acid and soy phytoestrogens, provides cardiopulmonary protection [110]. It raises the opening threshold of the mPTP under calcium overload in I/R myocardial cells, a protective effect partially reversible by the STAT3 inhibitor STATtic [111]. Intralipid also stimulates *STAT3* phosphorylation, promotes FAO, and enhances acetyl-CoA production, supporting mitochondrial health [112]. Propofol, a commonly used anesthetic, exerts anti-inflammatory and antioxidant effects [113]. It inhibits *STAT3* expression, reduces oxidative stress in hypoxia/reoxygenation (H/R)-induced cardiomyocytes, restores mitochondrial function, and protects cardiomyocytes [114]. Conversely, dexmedetomidine increases *JAK2/STAT3* pathway expression in H/R-induced cardiomyocytes, helping reduce mitochondrial apoptosis [115].

Additionally, FTY720, a food and drug administration (FDA)-approved sphingosine 1-phosphate (S1P) receptor inhibitor, may improve cardiac function [116]. It activates STAT3 and enhances mitochondrial respiration and ATP production in AC16 human cardiomyocytes [117].

### 5.3. Physical Therapy

Hypothermia has shown potential in slowing cardiac aging and treating heart disease. Studies indicate that hypothermia induces STAT3 phosphorylation at Tyr705, suppresses mPTP opening, preserves mitochondrial function, and reduces myocardial I/R injury [118].

**Table 1:** Treatments for combating cardiovascular diseases through the STAT3 protein.

Diseases	Treatments	Study Type(s)	Cell Line/Animal Model	Experimental Dose/Concentration (In Vitro/In Vivo [Administration Route(S)])	Regulation of STAT3 and Mitochondria	STAT3 Activators/Inhibitors	Refs
Atherosclerosis	Esculetin	In vitro/in vivo	Human aortic endothelial cells/ApoE^−/−^ mice	2.5 µM/0.5 mg/kg [p.o.]	Targets mitochondria, reduces p-STAT3 expression, lowers inflammation	Inhibitors	[87]
Acute myocardial infarction	Quercetin	In vivo	SD rats	50 mg/kg [p.o.]	Increases p-STAT3, inhibits ROS and MDA, reduces mPTP opening	Activators	[89]
Dox-induced cardiomyopathy	FL3	In vitro	Cardiomyocytes	100 nM	Increases p-STAT3 in mitochondria, reduces apoptosis	Activators	[91]
Dox-induced cardiomyopathy	7,8-dihydroxyflavone	In vitro/in vivo	Cardiomyocytes/kunming mice	100 μM/5 mg/kg [i.p.]	Increases p-STAT3, boosts ATP, restores MMP, inhibits ROS, restores mitochondrial complex I-IV	Activators	[93,94]
Dox-induced cardiomyopathy	Paeonol	In vitro/in vivo	Primary rat cardiomyocytes/SD rats	50 μM/75,150,300 mg/kg [p.o.]	Increases p-STAT3, promotes mitochondrial fusion via Mfn2	Activators	[96]
Diabetic cardiomyopathy	Rosmarinic acid	In vitro	Cardiomyocytes	5, 20, 50 mM	Increases p-STAT3, inhibits ROS, mPTP, cytochrome c release, and caspase-3	Activators	[98]
Diabetic cardiomyopathy	Taxifolin	In vitro/in vivo	Cardiomyocytes/C57BL/6	10 μg/mL,20 μg/mL and 40 μg/mL/25, 50, 100 mg/kg [p.o.]	Increases p-STAT3, restores MMP, reduces ROS	Activators	[99]
Cardiac hypertrophy	Taxifolin	In vitro	Chicken primary cardiomyocytes	0.5 μM	Inhibits p-STAT3, regulates mitochondrial ATP	Inhibitors	[100]
Diabetic cardiomyopathy	Punicalagin	In vitro/in vivo	Primary rat cardiomyocytes/SD rats	10 µM/30, 90 mg/kg [p.o.]	Increases p-STAT3, inhibits mitochondrial oxidative stress	Activators	[101]
Cardiac hypertrophy	Atorvastatin	In vitro/in vivo	Neonatal Rat Cardiomyocytes/C57BL/6J	10 µM/3 mg/kg [p.o.]	Inhibits p-STAT3, reduces FAO	Inhibitors	[75]
Dox-induced cardiomyopathy	Chemical mitochondrial uncouplers	In vitro	Neonatal Rat Cardiomyocytes	2, 0.05, 2 µM	Increases p-STAT3, boosts ATP production	Activators	[104]
Myocardial ischemia/reperfusion	Zinc	In vitro/in vivo	Cardiomyocytes/SD rats	1 µM/1 µM Zncl2 [perfusion]	Increases p-STAT3, inhibits mitochondrial ROS	Activators	[106]
Myocardial ischemia/reperfusion	Morphine	In vitro/in vivo	Cardiomyocytes/SD rats	0.1 µM/0.1 µM [perfusion]	Increases p-STAT3, inhibits mitochondrial ROS	Activators	[107]
Myocardial ischemia/reperfusion	Melatonin	In vitro/in vivo	Neonatal Rat Cardiomyocytes/SD rats	2 µM/5 µM [perfusion]	Increases p-STAT3, boosts mitochondrial SOD, inhibits H_2_O_2_, MDA, and mPTP	Activators	[109]
Myocardial ischemia/reperfusion	Intralipid	In vivo	SD rats	5 mL/kg/1% [perfusion]	Increases p-STAT3, inhibits mPTP, reduces Ca^2+^ overload, increases fatty acid oxidation	Activators	[111]
Myocardial ischemia/reperfusion	Propofol	In vitro	Cardiomyocytes	12.5, 25, 50 and 100 µM	Increases p-STAT3, inhibits MDA, boosts antioxidant enzyme activity	Activators	[112,114]
Myocardial ischemia/reperfusion	Dexmedetomidine	In vitro/in vivo	Cardiomyocytes/SD rats	1 μM/50 μg/kg [i.p.]	Increases p-STAT3, inhibits mitochondrial apoptosis	Activators	[115]
Myocardial ischemia/reperfusion	Hypothermia	In vitro/in vivo	Cardiomyocytes/SD rats	/	Increases p-STAT3, reduces mPTP, maintains mitochondrial function	Activators	[118]

In conclusion, targeted *STAT3* therapies, including natural compounds, clinical drugs, inhibitors, and physical therapy, regulate mitochondrial function and offer significant cardiovascular protection. However, the clinical translation of these therapeutic strategies remains confronted with substantial challenges. First, the existing evidence is largely derived from cell-based and animal models. *STAT3* exhibits context-dependent effects across different disease states, with distinct phosphorylation sites (Y705 vs. S727) and subcellular localization producing even opposing biological outcomes. This complexity indicates that simple activation or inhibition of *STAT3* is unlikely to represent a universally applicable therapeutic strategy and highlights the lack of precise, context-specific stratification. Second, many natural compounds suffer from poor bioavailability, pleiotropic targets, and poorly defined dose–response relationships, limiting their direct extrapolation to human applications. In addition, several clinical drugs modulate *STAT3* signaling in bidirectional or indirect manners, and their potential off-target effects and long-term safety profiles have not yet been systematically evaluated.

## 6. Conclusions and Future Perspective

This review examines the pivotal role of STAT proteins in regulating mitochondrial function during CVDs. Evidence clearly shows that STAT proteins significantly influence key mitochondrial processes in cardiac muscle cells, including ETC activity, membrane potential maintenance, ROS production, mPTP opening and closure, and mitochondrial dynamics.

Regarding therapeutic strategies, this review explores various interventions targeting the *STAT3*-mitochondria axis, including natural compounds, clinical drugs, specific inhibitors, and physical therapies. These approaches have shown promising potential in preclinical studies. However, clinical trials focusing on STAT-mitochondria pathways remain absent, posing a significant translational challenge. This challenge arises from the complex regulation of STAT proteins, which often exhibit contrasting dual roles in different CVD models. This functional duality complicates the development of targeted drugs. Furthermore, current research predominantly focuses on *STAT1* and *STAT3*, while the understanding of other STAT family members remains limited. The potential synergistic or antagonistic interactions among different STAT proteins in the same pathological context require urgent investigation.

Based on these considerations, this review proposes four key research directions for future studies: First, research should delve deeper into the dynamic regulation of STATs. Studies must systematically clarify how STATs regulate mitochondrial function across different disease stages, particularly the early versus late phases. A focus should be placed on tracking dynamic changes in subcellular localization, specifically the nucleus-to-mitochondria distribution ratio, and corresponding alterations in phosphorylation status. This approach may provide mechanistic insights into the functional duality of STATs. Second, the research perspective should expand to include organelle interactions. Future studies should explore how other organelles, such as the endoplasmic reticulum and lysosomes, influence mitochondrial function through STAT protein regulation. Understanding STAT functionality within the broader organelle interaction network is crucial. Third, increased attention should be given to non-canonical STAT proteins. Investigating *STAT2*, *STAT4*, *STAT5*, and *STAT6* is particularly important. Elucidating their specific mechanisms in mitochondrial regulation could uncover new potential therapeutic targets. Fourth, novel technologies should be integrated into mechanistic studies. Advanced methodologies, such as spatial single-cell transcriptomics, could enable the visualization of STAT protein activity and distribution within complex tissues, providing direct evidence to understand the spatiotemporal specificity of STAT functions in CVDs.

## Data Availability

No new data were created or analyzed in this study. Data sharing is not applicable to this article.

## Abbreviations

The following abbreviations are used in this manuscript:

CVDs	cardiovascular diseases
I/R	ischaemia–reperfusion
ROS	reactive oxygen species
STAT	signal transducer and activator of transcription
NTD	amino-terminal domain
CCD	coiled-coil domain
LD	linker domain
DBD	DNA-binding domain
TAD	transcriptional activation domain
JAK	Janus kinase
HGF	hepatocyte growth factor
PDGF	platelet-derived growth factor
EGF	epidermal growth factor
CSF-1	colony-stimulating factor 1
IL-6	interleukin-6
RTKs	receptor tyrosine kinases
GPCRs	G-protein-coupled receptors
Ang II	angiotensin II
mPTP	mitochondrial permeability transition pore
MMP	mitochondrial membrane potential
Drp1	dynamin-related protein 1
SIRPα	Signal Regulatory Protein Alpha
IL-4	interleukin-4
PGC1-α/β	peroxisome proliferator-activated receptor gamma coactivator 1-alpha/beta
VSMCs	vascular smooth muscle cells
C1P	ceramide-1-phosphate
H/R	hypoxia/reoxygenation
IL-35	interleukin-35
HDL	high-density lipoprotein
SAFE	SREBF Chaperone
IL-11	interleukin-11
CXCR4	chemokine receptor 4
Bcl-2	B-cell lymphoma 2
ISO	isoproterenol
DYRK1B	dual specificity tyrosine-(Y)-phosphorylation regulated kinase 1B
CPT1	carnitine palmitoyltransferase 1
FAO	fatty acid oxidation
RORα	RAR related orphan receptor α
DCM	dilated cardiomyopathy
DOX	doxorubicin
mtDNA	mitochondrial DNA
MFF	mitochondrial fission factor
CRT	calreticulin
HSP22	heat shock protein 22
PPARα	peroxisome proliferator-activated receptor α
TLR4	toll like receptor 4
NF-κB	nuclear factor kappa-B
MMP8	matrix metalloproteinase 8
FOXO1	forkhead box O1
IFN-γ	interferon-gamma
TNF-α	tumor necrosis factor-α
MDA	malondialdehyde
7,8-DHF	7,8-dihydroxyflavone
PAE	paeoniflorin
Mfn2	mitochondrial fusion protein 2
RA	rosmarinic acid
TAX	taxifolin
OPA1	optic atrophy 1
S1P	sphingosine 1-phosphate
Drp1	dynamin-related protein 1
ANP	atrial natriuretic peptide
BNP	b-type natriuretic peptide
MHC	myosin heavy chain
GP130	glycoprotein 130
PGC1α	peroxisome proliferator-activated receptor γ coactivator-1α
RAR	retinoic acid receptor
FL3	flavagline-inspired synthetic flavonoid
SOD	superoxide dismutase
FDA	food and drug administration
